# mGem: Horses for courses in mapping bacterial small RNA interaction networks

**DOI:** 10.1128/mbio.03082-25

**Published:** 2026-03-06

**Authors:** Simon L. Dove, Sahar Melamed

**Affiliations:** 1 Division of Infectious Diseases, Boston Children’s Hospital, Harvard Medical School 1811 , Boston, Massachusetts, USA; 2 Department of Microbiology and Molecular Genetics, Institute for Medical Research Israel-Canada, Faculty of Medicine, The Hebrew University of Jerusalem 26742, Jerusalem, Israel; Georgia Institute of Technology, Atlanta, Georgia, USA

**Keywords:** RIL-seq, iRIL-seq, sRNA, Hfq, ProQ, RNA–RNA interactions, post-transcriptional regulation

## Abstract

Over the past few decades, it has become increasingly clear that small RNAs (sRNAs) play important regulatory roles in bacteria. These RNA species often work in concert with RNA chaperones such as Hfq that facilitate their base-pairing with target mRNAs. The mapping of sRNA interaction networks through the identification of the RNA species that sRNAs base-pair with can therefore provide critical insights into the potential regulatory roles sRNAs play. Indeed, sRNA interaction networks can be complex, with some bacteria producing more than a hundred different sRNAs, each capable of targeting anywhere from a single mRNA to several hundred different mRNAs. Here, we highlight two high-throughput approaches, RNA interaction by ligation and sequencing and intracellular RNA interaction by ligation and sequencing, that enable transcriptome-wide identification of sRNA interaction partners. Both methods capture RNA–RNA interactions *in vivo* and exploit chaperone-associated RNA–RNA ligation to capture native sRNA–target duplexes, providing powerful and scalable strategies for determining bacterial sRNA interaction networks.

## PERSPECTIVE

## SMALL RNAs WORK IN CONCERT WITH RNA CHAPERONES AND PAIR WITH TARGET TRANSCRIPTS

Small RNAs (sRNAs) are bacterial RNA species that are anywhere between 50 and 500 nucleotides in length. Many sRNAs employ a short sequence (as few as 6 nucleotides), known as the seed sequence, to base-pair with target transcripts (1, 2). This base-pairing is often incomplete and, in gram-negative bacteria, is frequently mediated by the RNA chaperone Hfq (3). Hfq can bind simultaneously to both an sRNA and its mRNA target, resulting in activation or repression of translation, modulation of transcript stability, or both (1). For any given sRNA, of which there may be more than a hundred encoded by the bacterium (4), there can be as few as one, or as many as several hundred target transcripts (5). Post-transcriptional control by sRNAs can therefore have far-reaching effects.

## HOW DO YOU IDENTIFY THE TARGETS OF sRNAs?

Although RNA-sequencing approaches have revolutionized the identification of sRNAs in a plethora of bacteria (2), relatively little is known about the regulatory roles these sRNAs play because it is not known which RNA species they pair with. Moreover, because of the limited and incomplete base-pairing that occurs between the sRNA and its target, computationally predicting targets for sRNAs is challenging. Here, we focus on approaches for the global identification of targets of sRNAs that function by base-pairing with other RNA species. Our intention is not to provide an exhaustive review of all the elegant approaches that are available for doing this, but instead to highlight two related genome-wide approaches, one of which has seen widespread adoption across the field, and another that shows particular promise. These approaches essentially involve trapping paired RNA species on an RNA chaperone to identify all chaperone-bound sRNAs together with their target RNAs at once.

## RIL-seq: LINKING sRNAs TO TARGET RNAs

**Fig 1:**
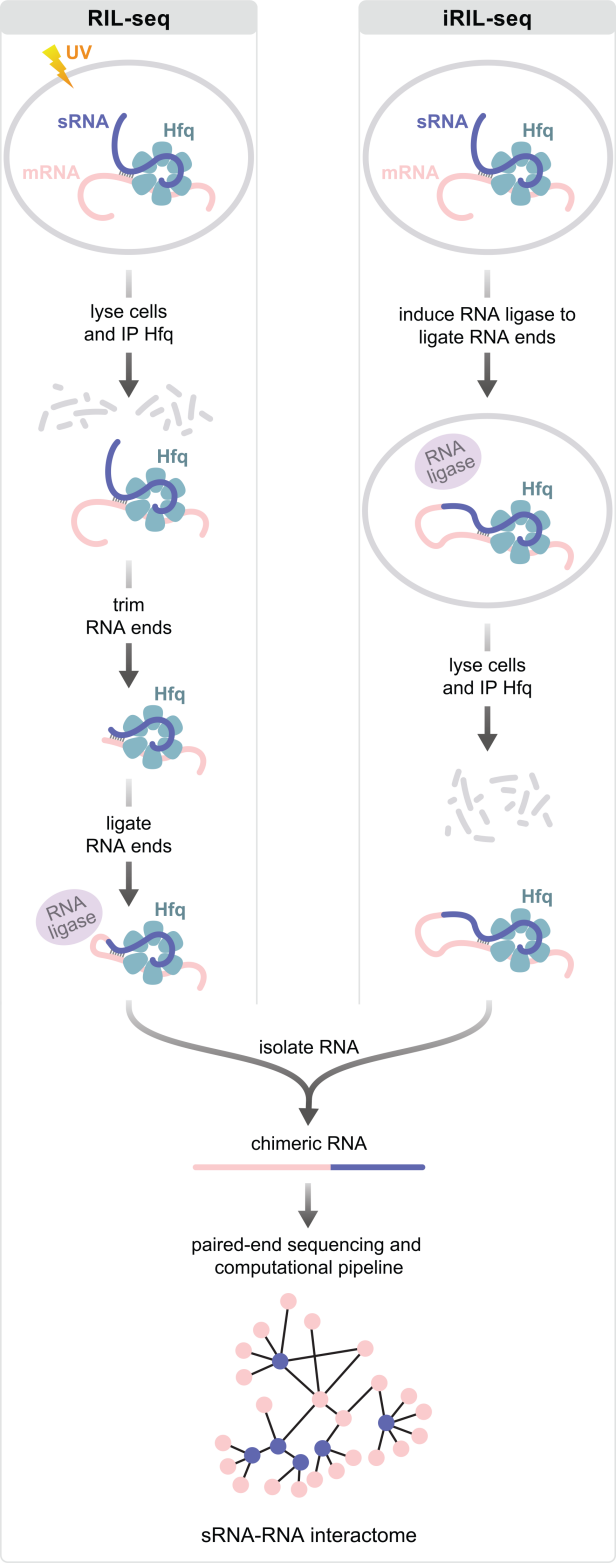
Schematic of RIL-seq (left) and iRIL-seq (right) approaches. Note that although mRNA species are depicted as paired with sRNAs on Hfq, other paired RNA species can be captured on Hfq using these approaches.

RIL-seq stands for RNA interaction by ligation and sequencing and is conceptually similar to the ligation-based approaches developed for identifying the targets of miRNAs in eukaryotes (6, 7). RIL-seq was first used to identify RNA–RNA interactions occurring on Hfq in *Escherichia coli*, with a focus on those involving sRNAs and their mRNA targets (8). In this approach, cells expressing an epitope-tagged version of Hfq are first irradiated with UV to cross-link sRNAs together with their paired targets on individual molecules of Hfq (Fig. 1). Next, cells are lysed, and Hfq, together with any associated RNA, is immunoprecipitated using an antibody against the tagged Hfq. The RNAs bound to Hfq are then trimmed with a ribonuclease which facilitates subsequent ligation by generating RNAs with monophosphates at their 5′ ends. Paired RNA species trapped on Hfq are then linked together *in vitro* with RNA ligase (Fig. 1) (8). The resulting chimeric RNA species often correspond to an sRNA ligated to its mRNA target and are identified by high-throughput sequencing. A dedicated bioinformatic pipeline is then used to identify chimeric RNA molecules by detecting sequencing fragments whose ends map to two distinct, noncontiguous genomic loci, indicative of RNA–RNA ligation events. Following alignment, candidate chimeras are subjected to stringent filtering and threshold criteria, such as minimum read support and enrichment over random ligation, to eliminate mapping artifacts and low-confidence events, thereby retaining only statistically significant and biologically meaningful RNA–RNA interactions (8, 9). Importantly, the UV cross-linking step in RIL-seq helps preserve Hfq–RNA complexes present *in vivo*, limiting RNA exchange after cell lysis and improving the fidelity of captured RNA–RNA interaction pairs (8).

The first RIL-seq study enabled the identification of targets for over a hundred Hfq-bound sRNAs in cells of *E. coli* grown under a variety of conditions (8). This study recapitulated most of the RNA–RNA interactome in *E. coli* that was known at that time and revealed many new sRNAs and targets. Indeed, RIL-seq, together with the related approach CLASH (10), provides a snapshot of all the detectable interactions involving Hfq-bound sRNAs at any point in time. The information obtained not only provides important insights into the potential regulatory roles of sRNAs but also points to where the base-pairing between an sRNA and its target occurs on the target transcript (5, 11). This can help prioritize follow-up studies to determine the extent to which a given sRNA exerts a regulatory effect on a particular target. RIL-seq can also provide clues as to the possible regulatory mechanisms sRNAs employ. Although many sRNAs pair with their target transcripts close to or overlapping the Shine-Dalgarno sequence, RIL-seq data revealed that sRNAs can pair with an mRNA in the body of the transcript (i.e., internal to coding sequence (CDS). This latter observation has led to the discovery that certain sRNAs can exert regulatory effects on protein activity by pairing deep within the CDS of an mRNA and potentially influencing the rate of translation (12).

Although we highlight the utility of RIL-seq in identifying mRNA species targeted by sRNAs, the approach also provides information on interactions between any RNA species that are occurring on a particular RNA chaperone such as Hfq, including both sRNA–sRNA interactions (see, for example, references 13, 14) and mRNA–mRNA interactions (15). In relation to the former, these sponging interactions between sRNAs on Hfq can modulate the availability or abundance of a given sRNA, thus modulating its activity, serving as an additional layer of regulation (16).

Since its initial use, RIL-seq has not only expanded our understanding of Hfq-bound sRNAs in *E. coli* under diverse conditions (17, 18), but has also been used successfully to identify the targets of Hfq-bound sRNAs in a variety of important environmental and pathogenic bacteria including enteropathogenic *E. coli* (19), *Salmonella enterica* (14), *Vibrio cholerae* (13), *Clostridium difficile* (20), *Pseudomonas aeruginosa* (21), *Klebsiella pneumoniae* (22, 23), *Caulobacter crescentus* (24), *Bordetella pertussis* (25), and *Acinetobacter baumannii* (26). Many of these studies involve bacteria in which little was known about sRNA-mediated regulation beforehand and provide important resources to those interested in whether a gene or subset of genes in a particular organism might be subject to control at the post-transcriptional level by one or more sRNAs. Key specific insights gained from many of these studies are reviewed elsewhere (5). Recently, RIL-seq has been applied to identify sRNA interaction networks occurring on Hfq in bacterial cells during prophage induction or phage infection, revealing roles played not only by sRNAs of the bacterial host, but also those of the phage, during these interactions (15, 27, 28).

Besides Hfq, RIL-seq can also be applied to the identification of RNA–RNA interactions occurring on another prominent RNA chaperone, ProQ, as was demonstrated in both *E. coli* and *V. cholerae* (29, 30). These studies led to the appreciation that ProQ can not only promote base-pairing between sRNAs and target mRNAs but also facilitate the pairing between different sRNA species (31). Applying RIL-seq to RNA-binding proteins other than Hfq or ProQ may reveal previously unrecognized roles for RNA-binding proteins as well as for sRNAs in bacterial regulation.

## FROM RIL-seq TO iRIL-seq: EXPANDING THE RNA–RNA INTERACTION TOOLKIT

The second genome-wide approach we highlight is referred to as intracellular RIL-seq (iRIL-seq) (32). This approach involves expressing an RNA ligase inside cells to ligate base-paired RNA species together (Fig. 1). In this regard, it resembles the GRIL-seq approach developed originally for identifying the targets of individual sRNA species (33) and requires a 5′ monophosphate, typically present on processed transcripts, on either the sRNA or its target to allow ligation. Following induction of the RNA ligase, cells are harvested, then lysed, and Hfq is immunoprecipitated to enable the identification of those chimeric transcripts bound to Hfq by high-throughput sequencing (Fig. 1) (32). Thus, unlike RIL-seq, iRIL-seq does not employ a UV cross-linking step, nor does it involve trimming of RNA species with ribonuclease following the IP of Hfq. On the one hand, with fewer sample processing steps, iRIL-seq may offer certain advantages over the standard RIL-seq approach. On the other hand, ectopic expression of the RNA ligase in iRIL-seq can interfere with cell growth and introduce stress (32–35), which could, in principle, influence which sRNAs are produced by the cell under a particular growth condition. Additionally, because RNA ligation in iRIL-seq occurs *in vivo*, the method presumably preferentially captures RNAs that are processed *in vivo*, resulting in a subset of RNA–RNA interactions within the broader regulatory network. Recent analyses in *E. coli* suggest that RIL-seq and iRIL-seq indeed produce comparable, albeit not identical results (34). All in all, RIL-seq and iRIL-seq can be viewed as complementary approaches, with RIL-seq enabling more comprehensive interactome mapping, while iRIL-seq provides a simpler, more streamlined experimental protocol that has been suggested might capture more transient interactions between sRNAs and their targets (34). It is worth emphasizing that both methods use the same RIL-seq computational pipeline (9, 32). To date, iRIL-seq has been used to identify RNA–RNA interactions occurring on Hfq (32, 34, 35), and it will be interesting to investigate how well it can capture interactions on other RNA chaperones, such as ProQ or ProQ-related proteins that harbor a FinO RNA-binding domain.

## FUTURE CHALLENGES

Both RIL-seq and iRIL-seq have provided significant insights into the regulatory roles of sRNAs in a variety of pathogenic and environmental bacteria (5, 35) grown as monocultures *in vitro*. How sRNA interaction networks are shaped in polymicrobial communities, where interspecies interactions and altered physiological states may substantially remodel RNA interactomes, is an open question. On a related note, how RNA–RNA interaction networks are shaped throughout growth in more complex and structured environments, such as those of the host, remains largely unexplored. The complementary features of RIL-seq and iRIL-seq may help address this gap, with RIL-seq suited for probing dynamic network changes over time and iRIL-seq potentially capturing interactions determined by a specific biogeographical location, provided RNA ligase induction can be linked to occupancy of a specific niche. Nonetheless, studying RNA interactomes in such complex settings presents significant challenges, including technical constraints associated with the use of UV irradiation or inducible systems. For example, in iRIL-seq, limited inducer accessibility under certain conditions may affect efficient expression of the RNA ligase. Despite these technical challenges, recent studies demonstrate that mapping sRNA interaction networks under host-associated environments is feasible using both of the approaches outlined here (36, 37).
